# Adjacent Forests Enhance Spontaneous Revegetation and Phylogenetic Similarity on Mountainous Roadside Slopes: Implications for Ecological Restoration and Forest Edge Management

**DOI:** 10.1002/ece3.73233

**Published:** 2026-03-22

**Authors:** Kun‐Rong Qin, Zi‐Zhuo Wang, Feng‐Ping Yang, Hua Qin, Xian‐Tao Peng, Hai‐Yang Wang

**Affiliations:** ^1^ School of Architecture and Design Chongqing College of Humanities, Science & Technology Chongqing China; ^2^ College of Horticulture and Landscape Architecture Southwest University Chongqing China

**Keywords:** adjacency effects, plant community assembly, revegetation, roadside slopes

## Abstract

Unstable roadside slopes in mountainous forested regions pose significant challenges for ecological restoration and the maintenance of forest ecosystem integrity. While passive (spontaneous) vegetation recovery offers a low‐cost and sustainable strategy, the underlying ecological drivers—particularly the role of adjacent forests and propagule sources—remain poorly understood. In this study, we examined 14 naturally restored roadside slopes and their adjacent forests in the subtropical mountains of southwestern China. Across 126 slope plots and 252 forest plots, we recorded 249 vascular plant species and assessed species composition, dispersal traits, and phylogenetic similarity between habitats. We found that 95.9% of slope species were native, with 80.0% exhibiting long‐distance dispersal traits, mainly through wind and birds. Approximately 65.3% of slope species were shared with adjacent forests, and the average phylogenetic similarity was relatively high (0.589), indicating that nearby forests act as key propagule sources. Structural equation modeling showed that species overlap and phylogenetic similarity were significantly influenced by forest species richness, forest cover, distance to core protected areas, and plant dispersal strategies. Phylogenetic dissimilarity between slope and forest communities was largely driven by lineage turnover, with nestedness contributing particularly in slope–forest pairs with close spatial proximity. These findings underscore the ecological importance of conserving adjacent forest habitats to facilitate spontaneous vegetation recovery on disturbed slopes. They also demonstrate that forest proximity, propagule pressure, and dispersal efficiency are critical to the success of passive restoration. From a management perspective, strategies should focus on maintaining forest connectivity and promoting landscape‐scale seed flow to enhance biodiversity recovery and slope stability in mountainous forest ecosystems.

## Graphical abstract

The native‐dominated vegetation on roadside slopes primarily originates from adjacent forests, with long‐distance dispersal traits enhancing spontaneous colonization in these disturbed environments. The species composition and phylogenetic similarity between slope and forest communities are significantly influenced by the adjacent forests (species composition and landscape structure) and the dispersal characteristics of propagules. Trategic conservation of adjacent forests and facilitation of seed dispersal across landscapes are key to achieving effective passive restoration of roadside slopes.

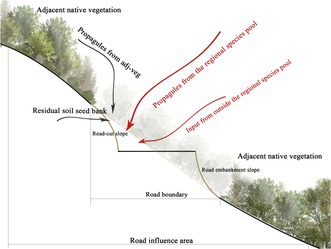

## 1 Introduction

Mountain ecosystems are globally acknowledged as biodiversity cradles, climate refugia, and extinction hotspots for countless species (Rahbek et al. 2019). However, the rapid expansion of linear infrastructure, particularly roads and railways, has increasingly disturbed these ecologically sensitive landscapes (Son et al. 2020). These anthropogenic developments fragment habitats, alter landforms, and lead to the widespread formation of steep, exposed roadside slopes. These artificial habitats are typically characterized by harsh abiotic conditions, such as shallow, nutrient‐depleted soils, intense surface runoff, and a lack of microsites suitable for plant establishment (Espigares et al. 2011; Azizi and Montazeri 2018). Mountainous roadside slopes are particularly vulnerable due to topsoil removal during construction, which depletes the soil seed bank and substantially limits regeneration potential (Arenas, Lázaro‐Lobo, et al. 2017). Under such circumstances, natural revegetation largely relies on propagule input from external sources, introducing considerable uncertainty into the restoration process and posing risks to regional biodiversity.

**FIGURE 1:**
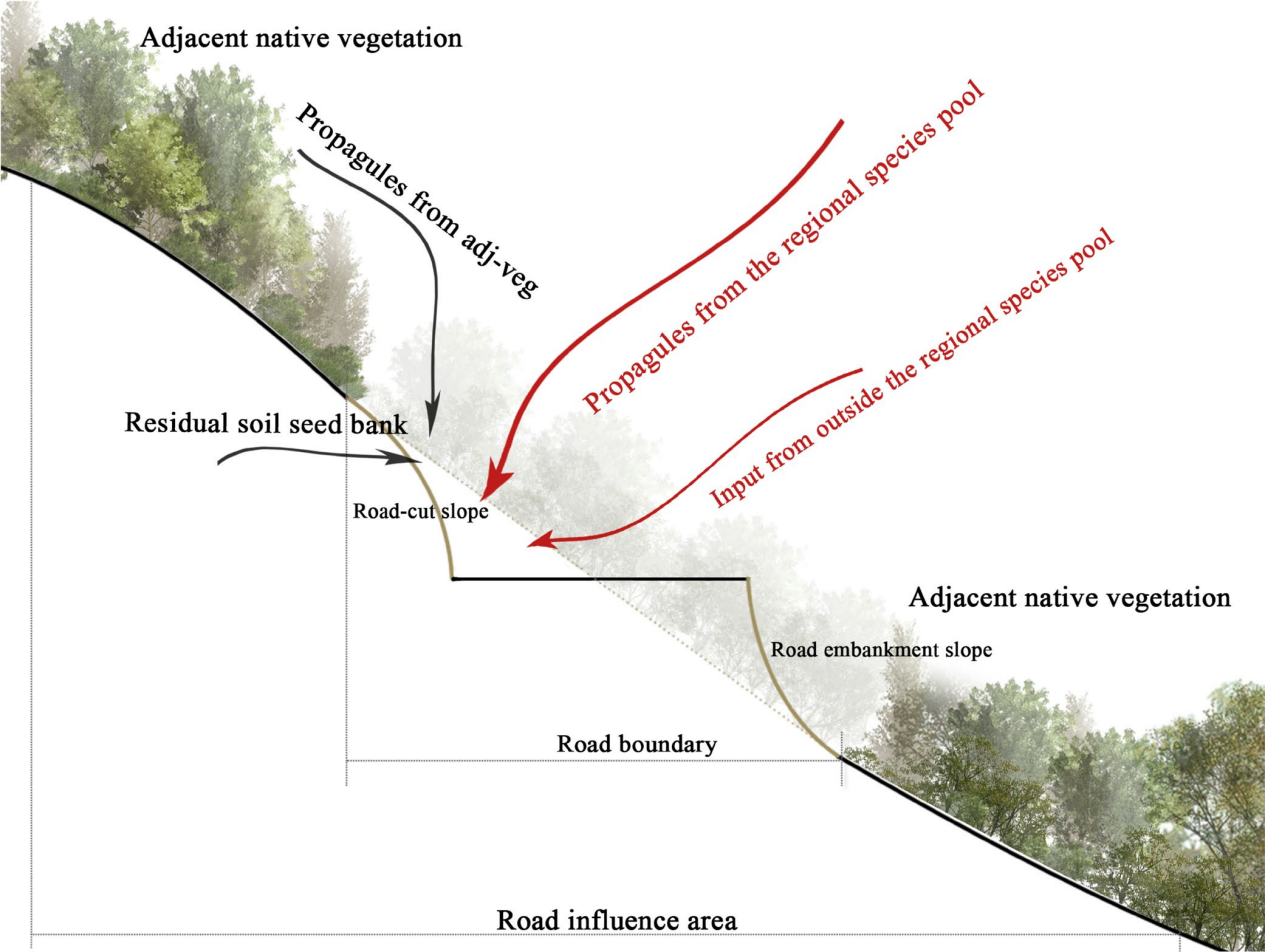
Conceptual illustration of potential propagule sources for self‐established vegetation on roadside slopes.

Although substantial progress has been made in understanding general revegetation dynamics, the specific sources and recruitment pathways of species during passive recovery remain poorly understood (Mourya et al. 2019). Colonizing species may originate from multiple non‐exclusive sources and pathways, including soil seed banks, propagule input from adjacent habitats, dispersal from the broader regional species pool, and road‐corridor–mediated long‐distance or human‐assisted inputs (Figure 1) (Bochet et al. 2007; Bochet and García‐Fayos 2015; Marteinsdóttir et al. 2018). Several studies have demonstrated that the structure of recovering communities is strongly influenced by propagule availability and dispersal traits in nearby undisturbed habitats (Son et al. 2020). For instance, Zhao et al. (2023) found that up to 66.1% of species in a restored pipeline corridor likely originated from adjacent vegetation, while De Villiers et al. (2003) reported that the regional species pool accounted for 30%–90% of aboveground species (De Villiers et al. 2003; Zhao et al. 2023). Roadside slopes may also function as entry points for ruderal or invasive species, particularly in disturbed sites with newly available ecological niches (Gelbard and Belnap 2003; Christen and Matlack 2006). These niches may support assemblages that show limited overlap with those of native forests. Moreover, dispersal limitations, especially for zoochorous species, can delay establishment in isolated or remote slopes (Cuperus et al. 1996; Verdu and GarciaFayos 1996). By contrast, wind‐dispersed (anemochorous) herbaceous species often dominate the early stages of succession due to high dispersal efficiency and prolific seed production (Nathan et al. 2002; Donath et al. 2003). As succession progresses, avian‐mediated seed dispersal becomes increasingly important for community assembly and species turnover (Howe and Miriti 2004; Sekercioglu 2006; Kleyheeg and van Leeuwen 2015). Growing empirical evidence suggests that revegetation is more successful when slopes are adjacent to well‐preserved forests (Zamora et al. 2010; Wang et al. 2013). These forests function as reservoirs of both taxonomic and functional diversity, offering abundant propagules to support species recovery (Herrera et al. 2011; de Torre et al. 2015). However, their influence is modulated by spatial and structural factors such as proximity, habitat connectivity, and surrounding vegetation composition (Zamora et al. 2010). Fragmented forests often exhibit reduced seed rain and diminished propagule pressure on nearby slopes (Poulsen et al. 2007; Leland Russell and Roy 2008).

Given the ecological vulnerability of mountainous regions and the growing pressures from infrastructure development, a better understanding of species origins, dispersal dynamics, and community assembly processes is urgently needed. In this study, we examine how adjacent forests influence spontaneous vegetation recovery on naturally restored roadside slopes in the subtropical mountains of southwestern China. Specifically, we address the following questions: (a) What are the primary sources and taxonomic compositions of species colonizing these slopes? (b) To what extent does adjacent forest vegetation contribute to slope community assembly? (c) How phylogenetically similar are slope and forest communities, and what factors shape this similarity? Answering these questions will improve our understanding of passive restoration mechanisms and inform landscape‐scale strategies for enhancing biodiversity and ecological resilience in forested mountain ecosystems.

## 2 Materials and Methods

### 2.1 Study Area

This study was conducted on the northwestern slope of Jilong Mountain, situated in the Dalou Mountain range within Wulong District, Chongqing Municipality, in southwestern China. The region is characterized by a subtropical humid monsoon climate with distinct seasonal variations in precipitation. The mean annual temperature ranges from 15°C to 18°C, while the average annual precipitation is between 1200 and –1400 mm, with approximately 1100 h of sunshine annually. The terrain is highly heterogeneous, featuring pronounced vertical zonation, rugged topography, and extensive forest cover. In recent years, extensive road construction in the region has resulted in the widespread formation of exposed roadside slopes. To minimize potential confounding effects from direct human activities, road segments distant from villages and other anthropogenic disturbances were selected. These segments pass through natural forests with varying levels of resource availability, including areas designated as nature reserves. All sampled slopes were undergoing natural recovery, with no artificial revegetation or engineering interventions having occurred since the roads were constructed. These conditions provide an ideal setting for assessing the role of adjacent forests in influencing the spontaneous vegetation recovery on roadside slopes.

### 2.2 Plot Design

**FIGURE 2:**
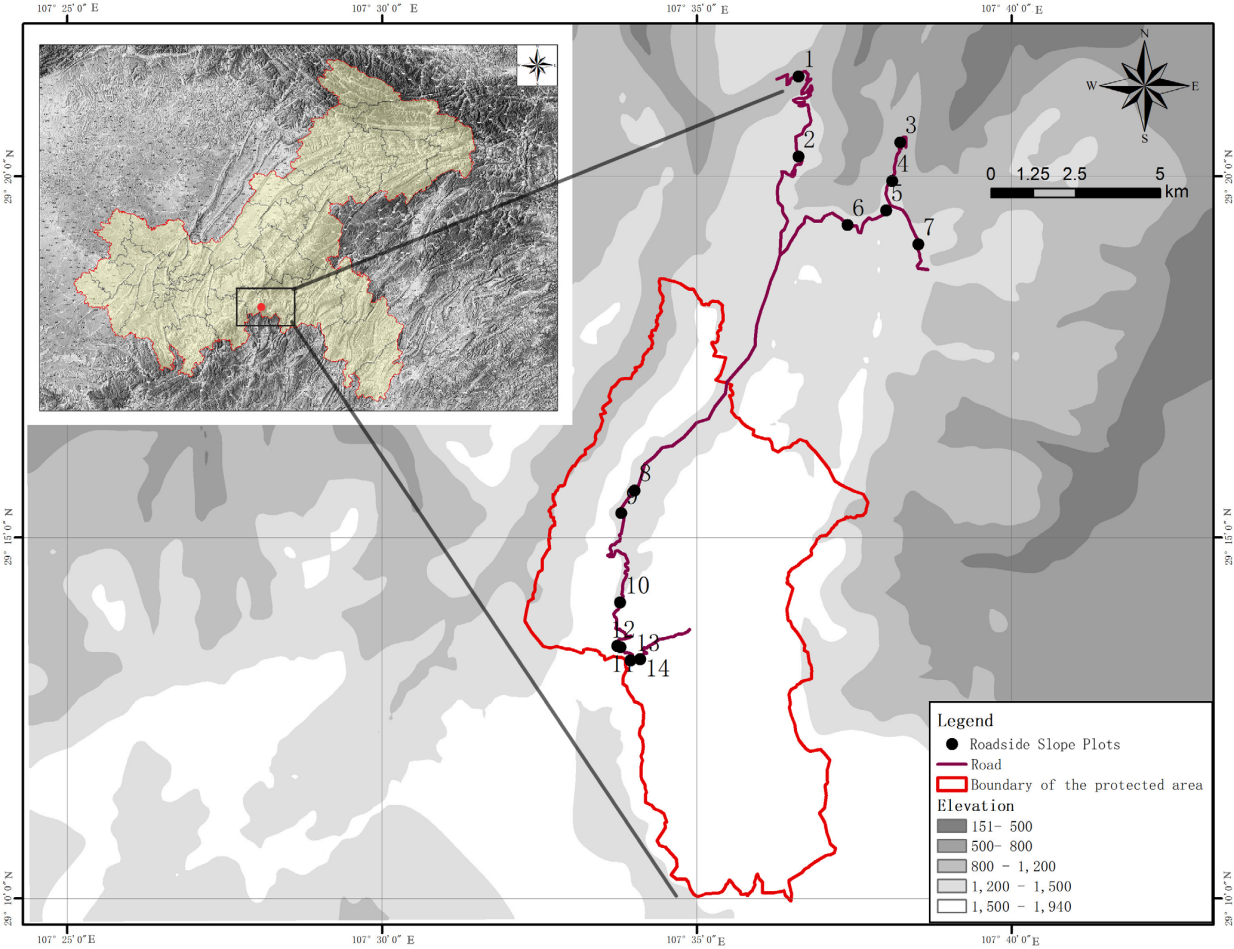
Spatial distribution of roadside slope plots in the study area.

To minimize the influence of environmental variation on species distribution, we selected 14 representative sampling sites within an elevation range of 1200–1600 m (Figure 2). All slopes were 5–10 m in height and had not experienced significant human disturbance. At each site, three belt transects (5 m wide) were established on the roadside slope perpendicular to the road to comprehensively survey plant community composition and naturally colonizing species. Along each transect, one shrub–herb plot (1 m × 3 m) was established at the upper, middle, and lower positions of the slope to capture vertical variation in species distribution and microhabitat conditions. To assess the influence of adjacent vegetation on slope communities, a corresponding 10 m × 30 m transect was established at the top of each slope. This transect was further divided into six 10 m × 5 m tree plots to systematically investigate the species composition and community structure of the adjacent forest. In total, nine shrub–herb plots were surveyed on each slope site, with a total sampling area of 27 m^2^ per site. In the adjacent forest, 18 tree plots were surveyed per site, with a total sampling area of 900 m^2^ per site. These areas met or exceeded commonly used standard plot sizes in vegetation surveys (25 m^2^ for shrubs and 400 m^2^ for forests). Overall, 126 slope plots and 252 forest tree plots were surveyed, and all vascular plant species were recorded in each plot. Because sampling areas differed between slope and forest habitats and both exceeded standard plot sizes, species richness values were not standardized by area for diversity comparisons. Subsequent analyses focused primarily on differences in species composition, species overlap, and phylogenetic relationships between habitats.

### 2.3 Field Survey and Data Collection

#### 2.3.1 Species Survey and Identification

A systematic inventory of vascular plant species was conducted within all sampled quadrats. For each species encountered, we recorded key ecological attributes, including taxonomic identity, individual abundance, plant height, percent cover, and growth status (i.e., vegetative or reproductive phase). Species identification was carried out in the field by experienced botanists, and voucher specimens were collected for subsequent verification. Taxonomic identification and nomenclature were confirmed using authoritative regional floras, primarily the *Flora of China* (https://www.iplant.cn/frps) and the *Identification Manual of Vascular Plants in Chongqing*. In addition, the compiled species list was cross‐referenced with two national databases: the *List of National Key Protected Wild Plants (2021 Edition)* and the *China Invasive Alien Species Information System* (https://www.iplant.cn/ias/), in order to identify legally protected species and detect the presence of non‐native invasive taxa within the study area. This rigorous protocol ensured the accurate classification of all plant species and provided a robust basis for subsequent analyses of community composition, biodiversity patterns, and successional dynamics.

#### 2.3.2 Environmental Variables Survey and Measurement

To evaluate the influence of environmental conditions on plant dispersal and community assembly, we quantified 11 environmental variables at each sampling site, encompassing topographic, spatial, soil, and landscape dimensions. Two topographic variables, aspect (ASP) and slope (SLO), were recorded due to their known effects on plant colonization and propagule dispersal. Spatial connectivity to regional species pools was assessed by measuring the distance to the nearest core zone of a nature reserve (DSP), which served as a proxy for the accessibility of propagules from protected habitats. Seven soil‐related variables were examined, including soil substrate thickness (THI) and soil moisture content (SMC). Soil moisture was measured at a depth of 10 cm using three replicates per site to ensure accuracy. In addition, five indicators of soil fertility were analyzed to evaluate nutrient status: soil organic carbon (SOC), available nitrogen (SAN), available phosphorus (SAP), available potassium (SAK), and pH. Soil samples were collected from the 0 to 20 cm layer, air‐dried, and sieved through a 0.15 mm mesh prior to chemical analysis. At the landscape level, forest proportion (For.P) was calculated as the percentage of forested land within a 1 km radius of each site, based on high‐resolution land cover data. Soil chemical properties were determined using standard analytical methods. Soil organic matter (SOM) was measured by the Walkley–Black dichromate oxidation method (Walkley and Black 1934). Available nitrogen (AN) was determined using the Kjeldahl digestion procedure (Bremner and Mulvaney 1982). Available phosphorus (AP) was extracted with the Olsen method and quantified colorimetrically (Olsen and Sommers 1982). Exchangeable potassium (AK) was extracted using ammonium acetate and analyzed by flame photometry (Knudsen et al. 1982). Soil pH was measured in a 1:1 soil‐to‐water suspension using a calibrated pH meter (Thomas 1996). Rigorous quality control was applied during both sampling and analysis to ensure consistency and accuracy across all sites.

#### 2.3.3 Determination of Slope Recovery Time

The natural recovery time of roadside slopes is a key variable for assessing species dispersal dynamics and community succession. To ensure objectivity and accuracy, recovery time estimates were cross‐validated using official infrastructure records, historical satellite imagery, and locally sourced information. These data were supplemented by interviews with local officials and long‐term residents knowledgeable about the road construction history in the region, providing temporal context not captured in formal records. To further refine temporal resolution, multi‐temporal remote sensing data were interpreted, including archival imagery from Google Earth and Landsat satellites, to identify construction dates and subsequent vegetation changes. The recovery time at each site was ultimately defined as the number of years since road construction completion.

### 2.4 Data Analysis

#### 2.4.1 Species Composition Analysis

All vascular plant species recorded in roadside slope and adjacent forest communities were systematically classified based on field surveys and published literature. Species were assigned to functional groups according to two criteria: growth form and dispersal mode. Growth forms included herbaceous vines, woody vines, annual/biennial herbs, perennial herbs, shrubs, and trees. Dispersal modes were categorized as animal‐mediated (zoochory), wind‐mediated (anemochory), gravity‐based (barochory), and self‐dispersal (autochory). Stacked bar charts were generated to visualize species composition, growth form distribution, and dispersal strategy across slope and forest habitats. These visualizations facilitated the comparison of community structure and allowed for the identification of both shared patterns and distinct differences between the two habitat types.

#### 2.4.2 Phylogenetic Diversity, Similarity, and Differentiation

To evaluate differences in phylogenetic diversity and community structure between roadside slopes and adjacent forests, a set of commonly used phylogenetic metrics was calculated using the “picante” package in R. These included phylogenetic diversity (PD), species richness (SR), phylogenetic species variability (PSV), phylogenetic species richness (PSR), phylogenetic species evenness (PSE), phylogenetic species clustering (PSC), the net relatedness index (NRI), and the nearest taxon index (NTI). One‐way analysis of variance (ANOVA) was employed to test for significant differences between habitat types. A species‐level phylogenetic tree was constructed using the “get_tree()” function from the “rtrees” package, based on the regional species pool. Pairwise phylogenetic similarity between communities was calculated using the “phylosor()” function in the “ape” package. Three types of pairwise comparisons were examined: between each slope and its adjacent forest (AS_AF), between non‐adjacent slopes and forests (S_F), and among slope communities (S_S). These analyses allowed for the assessment of phylogenetic similarity and community differentiation along both spatial and habitat gradients.

#### 2.4.3 Drivers of Shared Species Richness and Phylogenetic Similarity

To identify the ecological drivers of shared species richness and phylogenetic similarity between roadside slopes and adjacent forests, Partial Least Squares Path Modeling (PLS‐PM) was applied following the framework of Sanchez (Sanchez 2013). The analysis was conducted using the “plspm()” function in the “plspm” R package. Six latent variables were defined, each represented by multiple observed indicators. These constructs included topographic conditions, recovery time, soil properties, species richness of adjacent forests, dispersal mode structure, and the response variables: richness of shared species and the phylogenetic similarity index between slope and forest communities.

The model was constructed to quantify both direct and indirect effects among ecological factors, thereby clarifying the relative influence of abiotic, biotic, and landscape drivers on community convergence. Model performance was evaluated using the Goodness‐of‐Fit (GOF) index, with values above 0.6 considered acceptable according to current ecological modeling standards (Wang et al. 2024). Indicator loadings were assessed to determine the contribution of each observed variable to its latent construct; loadings exceeding 0.7 were retained as significant (Hulland 1999). The model was iteratively refined to improve structural clarity and explanatory power.

#### 2.4.4 Phylogenetic Differentiation and Its Ecological Drivers

To quantify phylogenetic dissimilarities among communities across sampling sites, pairwise phylogenetic beta diversity (β‐diversity) was calculated using the “phylo.beta.pair ()” function in the “betapart” package. This approach decomposes β‐diversity into two additive components: phylogenetic turnover, representing species replacement, and phylogenetic nestedness, indicating richness differences based on evolutionary lineage. To visualize the relative contributions of these components, ternary plots were produced using the “triangle.plot ()” function from the “ape” package. These plots provided an interpretable graphical representation of the balance between turnover and nestedness in shaping overall phylogenetic β‐diversity. Subsequently, Partial Least Squares Path Modeling (PLS‐PM) was applied to identify the primary ecological drivers of phylogenetic differentiation. Particular emphasis was placed on evaluating the roles of dispersal limitation, environmental filtering, and landscape‐level processes in driving community divergence.

All continuous variables were standardized using *Z*‐score transformation prior to analysis to ensure comparability across different scales. Statistical analyses and visualizations were performed in R version 4.0.3.

## 3 Results

### 3.1 Species Composition

A total of 249 vascular plant species, representing 98 families and 152 genera, were recorded across the 14 sampling sites. Among these, 202 species (88 families, 142 genera) were found in adjacent forest plots, whereas only 98 species (46 families, 75 genera) were recorded on roadside slopes. This marked difference reflects substantial variation in species richness and taxonomic composition between the two habitat types. In forest communities, ten plant families each contained more than five species, collectively accounting for 44.1% of the total species pool. This indicates a relatively even and taxonomically dispersed community structure. By contrast, only four families on roadside slopes exceeded five species, contributing 38.8% of the total species count, which suggests a more taxonomically concentrated assemblage. Native species were predominant in the slope environment, with 94 indigenous species identified. Additionally, 4 alien invasive species were recorded. Among the native flora, three species were identified as regionally endemic to the slope habitats, and one nationally protected species was also documented. Summary statistics for each sampling site, together with the dominant species of forest and slope habitats, are provided in Table 1.

**TABLE 1:** Basic information of sampling points between slope and adjacent vegetation.

Plot	Slop			Adjacent vegetation		
	Slope (°)	Cover (%)	Dominant species	Slope (°)	Cover (%)	Dominant species
R1	77	67	*M. sin* + *C. ind* + *P. vit*	28	100	*C. lan*‐*V. ero*‐*R. bue*
R2	74	42	*R. rch* + *L. per* + *L. rub*	23	100	*C. lan*‐*V. set*‐*R. bue*
R3	77.3	63	*P. mas* + *R. sim* + *M. sin*	50	100	*P. mas*‐*R. bac*‐*O. und*
R4	73.5	69	*M. sin* + *D. dch* + *C. sti*	29	100	*C. lan*‐*F. het*‐*R. bue*
R5	39	84	*M. sin* + *P. mas* + *R. rch*	31	100	*C. lan‐S. hir‐R. bue*
R6	71	25	*L. per* + *V. gry* + *W. jap*	55	100	*P. con‐M. dav‐S. sto*
R7	43	89	*M. sin* + *Q. ser* + *P. aqu*	32	100	*C. lan*‐*P. spi*‐*R. bue*
R8	73	60	*C. acu* + *V. ero* + *A. sub*	46	100	*C. jap*‐*E. loq*‐*D. dch*
R9	54.3	55	*R. rch* + *P. vil* + *A. his*	45	100	*C. jap‐R. sim‐D. dch*
R10	50	81	*R. coe* + *F. spa* + *R. aur*	24	100	*L. ped*‐*F. spa*‐*D. ery*
R11	88.3	89	*H. str* + *R. bac* + *A. sub*	24	100	*C. car*‐*E. loq*
R12	64.5	56	*C. jap* + *C. acu + H. xan*	26	100	*C. lan*‐*C. pit*‐*M. nan*
R13	71.6	52	*H. str* + *P. vil* + *A. his*	24	100	*C. tur‐E. loq*‐*C. acu*
R14	52	90	*F. spa* + *S. amp* + *A. ela*	46	100	*Q. gla*‐*Q. gla*‐*F. spa*

*Note:* Species names are abbreviated as the first letter of the genus followed by the first three letters of the specific epithet.

Abbreviations: *A. ela*, *Aralia elata; A. his*, *Arthraxon hispidus; A. sub*, *Aster trinervius*; *C. acu*, *Cyclosorus acuminatus; C. car, Castanopsis carlesii; C. ind*, *Chrysanthemum indicum; C. jap*, *Cryptomeria japonica; C. lan*, *Cunninghamia lanceolata; C. lan*, *Cunninghamia lanceolata; C. pit*, *Camellia pitardii; C. sti*, *Carex stipitinux. C. tur*, *Carpinus turczaninowii; D. dch*, *Diplopterygium chinense; D. ery*, *Dryopteris erythrosora; E. loq*, *Eurya loquaiana; F. het*, *Ficus heteromorpha; F. spa*, *Fargesia spathacea; H. str*, *Hydrangea strigosa; H. xan*, *Hydrangea xanthoneura; L. per*, *Lolium perenne; L. rub*, *Litsea rubescens; M. dav*, *Metapanax davidii; M. nan*, *Machilus nanmu; M. sin*, *Miscanthus sinensis; O. und*, *Oplismenus undulatifolius; P. aqu*, *Pteridium aquilinum; P. con*, *Prunus conradinae; P. mas*, *Pinus massoniana; P. spi*, *Prunus spinulosa; P. vil*, *Patrinia villosa; P. vit*, *Pteris vittata; Q. gla*, *Quercus glauca; Q. ser*, *Quercus serrata; R. aur, Rhododendron auriculatum; R. bac*, *Rhododendron bachii; R. bue*, *Rubus buergeri; R. coe*, *Rhododendron coeloneurum; R.rch*, *Rhus chinensis; R. si*, *Rhododendron simsii; S. hir*, *Spiraea hirsuta; S. sto*, *Saxifraga stolonifera; V. ero*, *Viburnum erosum; V. ero*, *Viburnum erosum; V. gry*, *Viola grypoceras; V. set*, *Viburnum setigerum; W. jap, Woodwardia japonica*.

### 3.2 Growth Form Composition and Dispersal Mode Differences

On roadside slopes, a total of 16 tree species (including seedlings and saplings), 30 shrubs, 4 annual or biennial herbs, 44 perennial herbs, 3 woody lianas, and 1 herbaceous vine were recorded. In contrast, adjacent forest plots contained 51 tree species, 76 shrubs, 8 annual/biennial herbs, 58 perennial herbs, 6 woody lianas, and 3 herbaceous vines. Excluding rarely recorded species, on the slopes, all species other than trees included mature reproductive individuals, whereas tree species occurred only in vegetative stages. In the forest, reproductive individuals were observed for all life forms.

Analysis of growth form composition revealed that slope communities were primarily composed of perennial herbs (44.9%) and shrubs (30.6%), which together accounted for 75.5% of all slope species. In forest communities, the proportion of shrubs was similar (31.2%), while perennial herbs were less prevalent (28.7%), and tree species contributed a substantially higher proportion, showing an increase of 8.9% compared with slope habitats. These patterns suggest that slope communities are characterized by herb–shrub dominance, whereas forest communities maintain a more structurally developed tree layer.

Regarding dispersal strategies, 119 species were identified as wind‐dispersed, 66 as animal‐dispersed (predominantly by birds), 35 as gravity‐dispersed, and 11 as mechanically dispersed. Approximately 80% of all species exhibited traits indicative of long‐distance dispersal potential. In slope habitats, anemochorous species comprised 61.2% of the total—nearly twice their proportion in adjacent forests. In contrast, zoochorous species (20.4%) occurred at roughly half the frequency observed in forests (39.6%). Collectively, these patterns suggest that slope habitats are preferentially colonized by pioneer species possessing highly efficient dispersal mechanisms.

**FIGURE 3:**
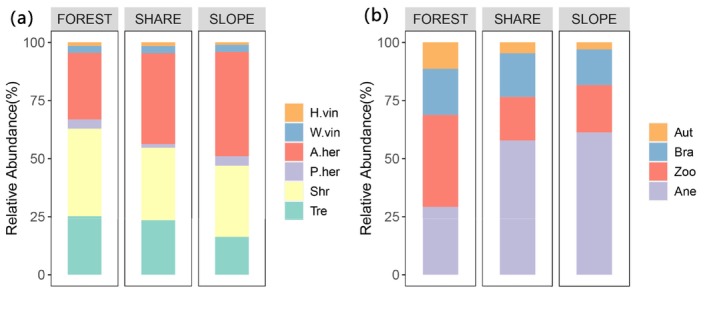
Proportional composition of growth forms and dispersal modes in slope, forest, and shared plant species. FOREST represents the adjacent forest community, SHARE refers to species shared between the roadside slope and adjacent forest, and SLOPE indicates the roadside slope plant community. H.vin, W.vin, A.her, P.her, Shr, and Tre represent herbaceous vines, woody vines, annual/biennial herbs, perennial herbs, shrubs, and trees, respectively. Aut, Bra, Zoo, and Ane represent autochory, barochory, zoochory, and anemochory, respectively.

A total of 64 species were found to be shared between roadside slopes and adjacent forests. Their growth forms and dispersal modes were analyzed separately. The functional composition of these shared species displayed intermediate characteristics between the two habitats but more closely resembled the profile observed in slope communities (Figure 3). This functional bias toward slope‐adapted traits reflects the combined effects of environmental filtering and dispersal limitation in shaping slope community assembly.

### 3.3 Phylogenetic Diversity and Similarity

**FIGURE 4:**
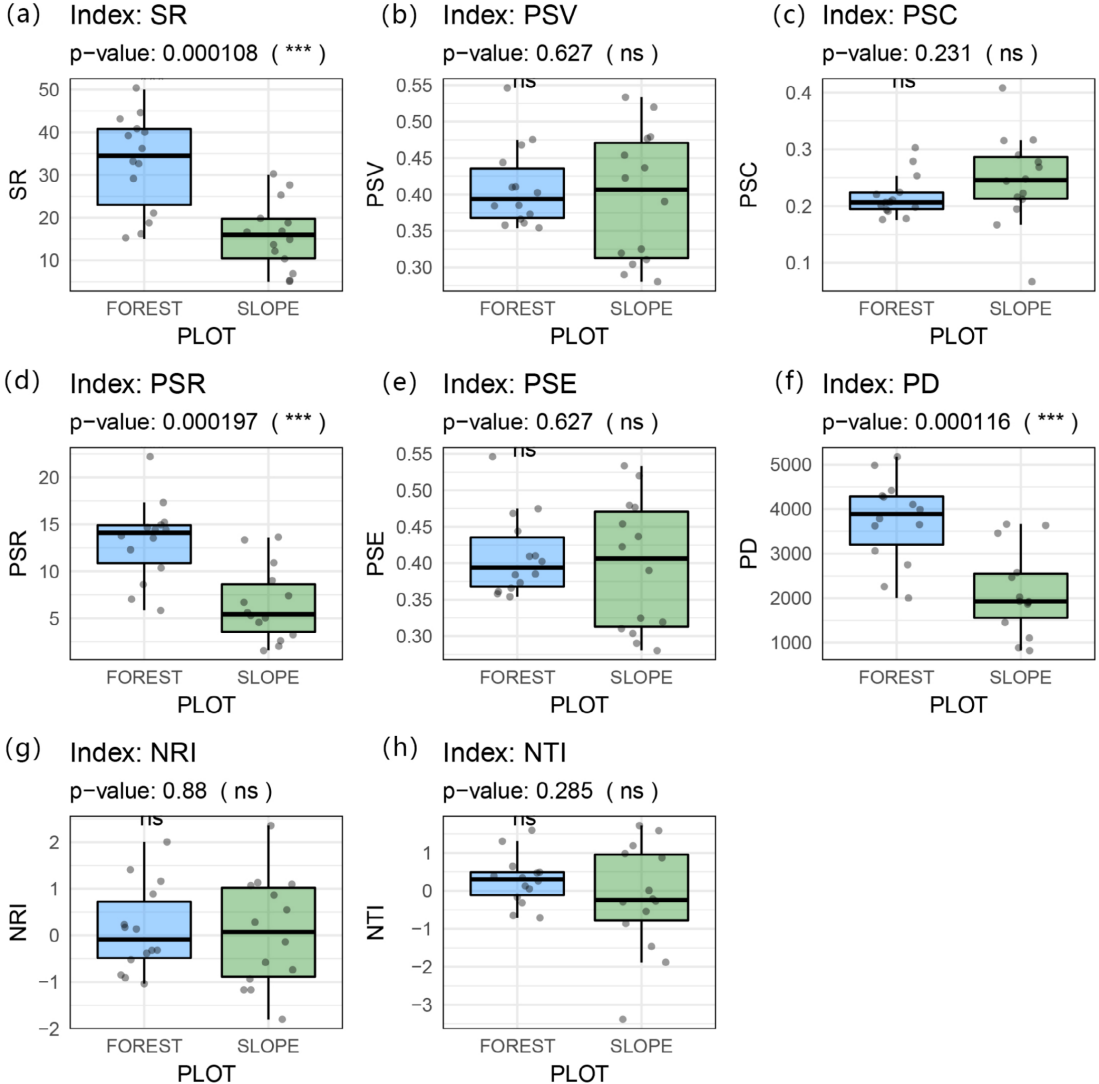
Differences in phylogenetic diversity between roadside slope and adjacent forest plant communities. (a) species richness (SR); (b) phylogenetic species variability (PSV); (c) phylogenetic species clustering (PSC); (d) phylogenetic species richness (PSR); (e) phylogenetic species evenness (PSE); (f) phylogenetic diversity (PD); (g) the net relatedness index (NRI); (h) the nearest taxon index (NTI). ns = not significant; ˙*p* < 0.1; *p < 0.05; **p < 0.01; ***p < 0.001.

Significant differences in phylogenetic diversity were observed between roadside slopes and adjacent forests (*p* < 0.05; Figure 4). Forest communities consistently exhibited higher species richness (SR), phylogenetic species richness (PSR), and phylogenetic diversity (PD) compared to slope communities. These results suggest that forest habitats support a broader phylogenetic spectrum and a more evolutionarily dispersed species pool, reflecting a richer evolutionary history.

In contrast, indices of phylogenetic structure—such as phylogenetic species variability (PSV), phylogenetic species evenness (PSE), phylogenetic species clustering (PSC), the Net Relatedness Index (NRI), and the Nearest Taxon Index (NTI)—showed no significant differences between slope and forest communities. This indicates that, despite lower species richness and narrower phylogenetic breadth in slope habitats, their internal phylogenetic structure did not exhibit a strong tendency toward clustering or overdispersion. In other words, slope communities were neither phylogenetically convergent nor divergent, suggesting a largely neutral or stochastic assembly pattern regarding evolutionary relatedness.

**FIGURE 5:**
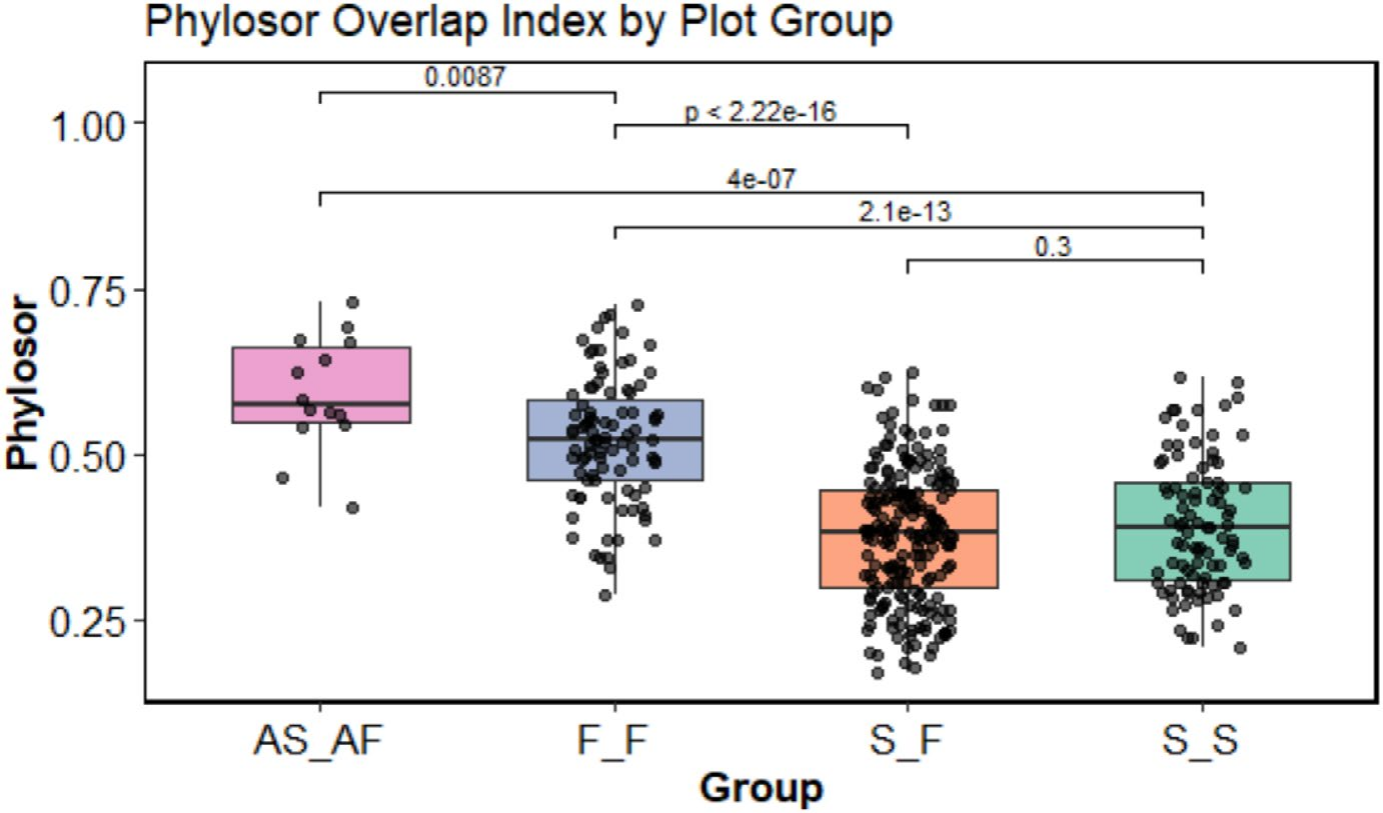
Analysis of variance in phylogenetic similarity indices across different plant community groupings. AS_AF refers to slopes and their adjacent forests; S_F indicates non‐adjacent slopes and forests; S_S represents comparisons among slope communities; and F_F denotes comparisons among forest communities.

To further investigate phylogenetic relationships among communities, we calculated the PhyloSor index for all pairwise plot combinations. Four community pairings were considered: slope–slope (S_S), slope–non‐adjacent forest (S_F), slope–adjacent forest (AS_AF), and forest–forest (F_F). Among these, slope plots and their directly adjacent forests (AS_AF) exhibited the highest mean phylogenetic similarity (PhyloSor = 0.589), which was significantly greater than all other pairings (*p* < 0.05; Figure 5). Despite lower species richness and diversity on slopes, the phylogenetic overlap with adjacent forests remained relatively high. This suggests that slope communities are predominantly composed of species from clades already present in neighboring forests, indicating that early‐stage slope vegetation assembly is primarily driven by recruitment from adjacent forest species pools. Additionally, anthropogenic disturbances have not yet caused strong phylogenetic divergence at the community level.

### 3.4 Drivers of Species Similarity Between Slopes and Adjacent Forests

**FIGURE 6:**
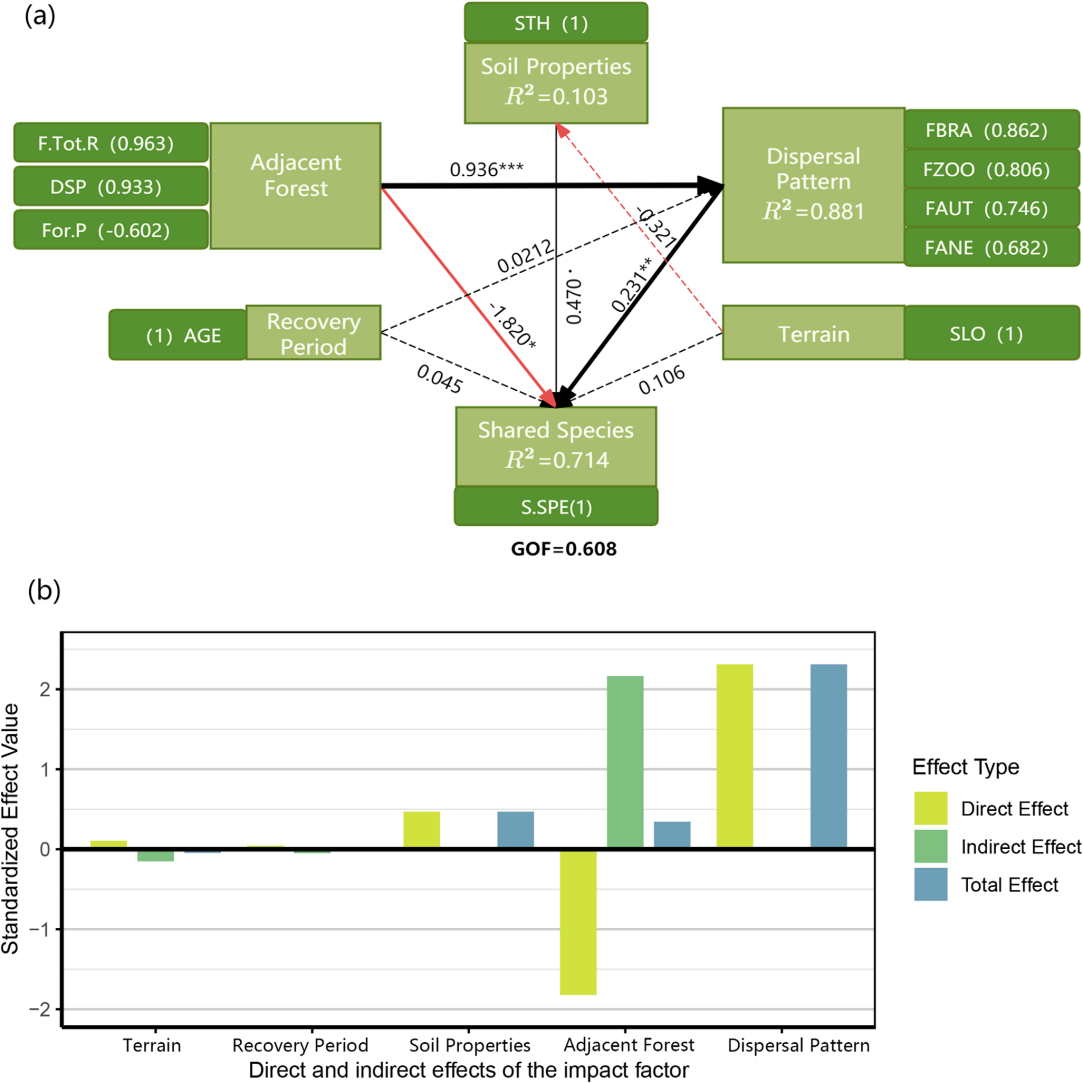
(a) The PLS‐PM model revealing the direct and indirect effects of adjacent forest, soil properties, species dispersal modes, restoration age, topography, and other factors on the shared species richness between roadside slopes and adjacent forests. Black lines indicate positive correlations, while red lines represent negative correlations. Solid lines denote significant relationships with significance levels as follows: ˙*p* < 0.1; *p < 0.05; **p < 0.01; ***p < 0.001. Dashed lines indicate non‐significant relationships. The thickness of the lines reflects the strength of causal relationships, accompanied by standardized path coefficients; (b) Bar chart showing the direct, indirect, and total effects of each factor.

The optimal structural model explained 71.40% of the variance in shared species richness (*R* ^2^ = 0.714; GOF = 0.608; Figure 6), demonstrating strong explanatory power and confirming that both environmental conditions and dispersal traits are critical factors in shaping early‐stage slope communities.

Within the model, adjacent forest characteristics and plant dispersal patterns were identified as the primary drivers of species overlap. Adjacent forest characteristics had a significant negative effect on shared species richness (path coefficient = −1.820, *p* < 0.05). This effect was mediated by three structural indicators: forest species richness (F.Tot.R; loading = 0.963), distance to the core species pool (DSP; loading = 0.933), and forest cover proportion (For.P; loading = −0.602). In contrast, dispersal patterns showed a significant positive effect on shared species richness (path coefficient = 0.231, *p* < 0.01). Among dispersal modes, gravity dispersal (FGRA; loading = 0.862) and animal‐mediated dispersal (FZOO; loading = 0.806) had the strongest contributions, highlighting the role of dispersal processes in facilitating species exchange between slopes and nearby forests.

Soil physicochemical properties exhibited a non‐significant positive effect (path coefficient = 0.470, *p* < 0.1), suggesting a weak influence of environmental filtering. Neither topographic factors (Ter) nor restoration age had significant effects, indicating that, in steep mountainous environments, the richness of shared species is more strongly influenced by input from well‐adapted external species pools than by local site conditions or the time since disturbance.

**FIGURE 7:**
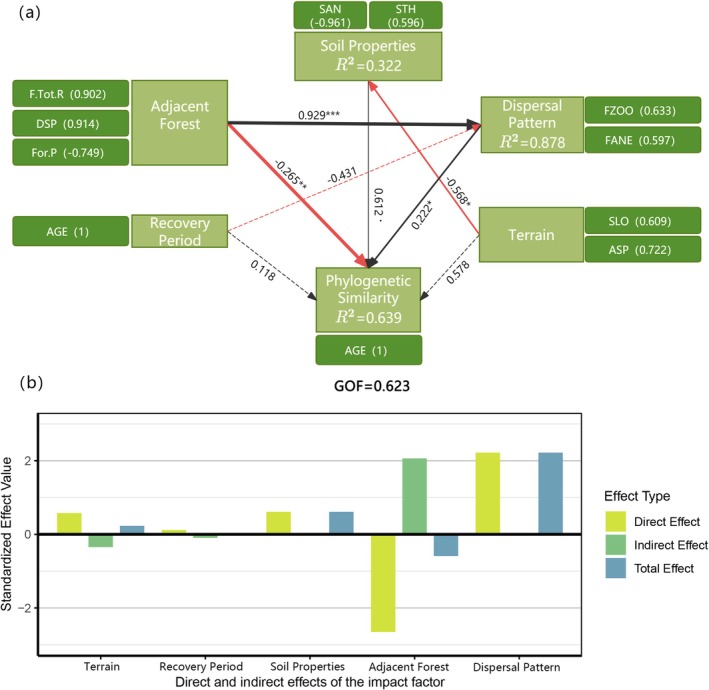
(a) The PLS‐PM model revealing the direct and indirect effects of adjacent forest, soil properties, species dispersal modes, restoration age, topography, and other factors on the phylogenetic similarity between roadside slopes and adjacent forests. Black lines indicate positive correlations, while red lines represent negative correlations. Solid lines denote significant relationships with significance levels as follows: ˙*p* < 0.1; *p < 0.05; **p < 0.01; ***p < 0.001. Dashed lines indicate non‐significant relationships. The thickness of the lines reflects the strength of causal relationships, accompanied by standardized path coefficients; (b) Bar chart showing the direct, indirect, and total effects of each factor.

In the model evaluating phylogenetic similarity between roadside slopes and adjacent forests, we identified a consistent structure of controlling factors. Dispersal mode (path coefficient = 0.222, *p* < 0.05) and adjacent forest characteristics (path coefficient = −0.265, *p* < 0.01) emerged as the dominant causal pathways, collectively explaining 63.9% of the variation in phylogenetic similarity (*R* ^2^ = 0.639; GOF = 0.623, Figure 7). The positive influence of dispersal mechanisms was primarily attributable to zoochory (FZOO, loading = 0.633) and anemochory (FANE, loading = 0.597), suggesting that these long‐distance dispersal vectors promote species co‐occurrence across spatially separated habitats, thereby enhancing phylogenetic congruence between plant assemblages on slopes and those in adjacent forests. In contrast, adjacent forest characteristics exhibited a significant negative association with phylogenetic similarity (path coefficient = −0.265, *p* = 0.00907). This relationship was mediated by three principal structural variables: forest species richness (F.Tot.R, loading = 0.902), spatial distance to the core species pool (DSP, loading = 0.914), and forest cover proportion (For.P, loading = −0.749). These results imply that higher biodiversity and increased spatial isolation of adjacent forests, together with lower forest cover, may constrain phylogenetic similarity, potentially due to environmental filtering or limitations in effective dispersal.

### 3.5 Drivers of Species Similarity Between Slopes and Adjacent Forests

**FIGURE 8:**
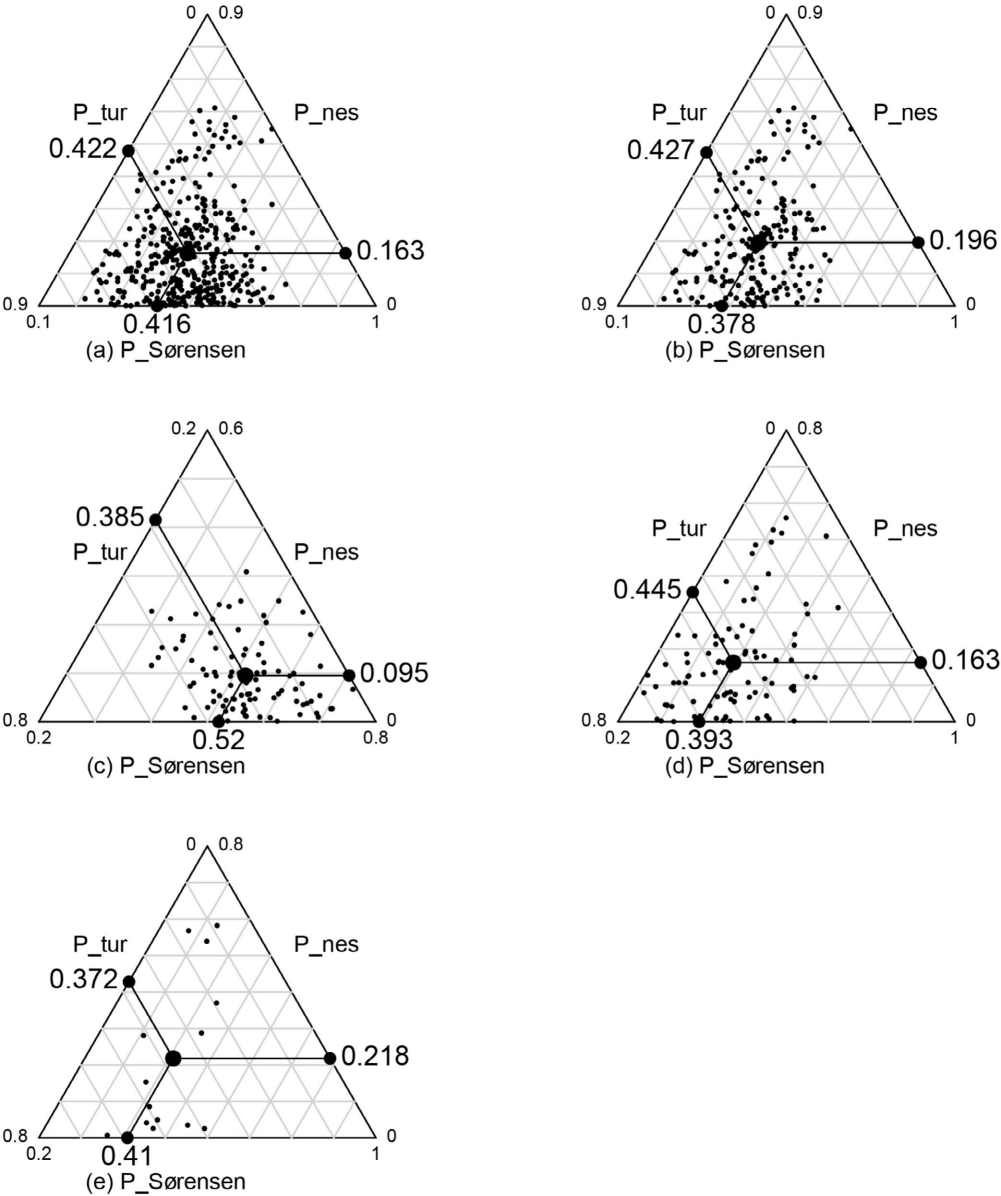
Triangular plot of pairwise indices (Sorensen similarity, turnover, and nestedness) between slope and forest plots. (a) All plot pairs (b) Non‐adjacent forest‐slope plot pairs; (c) Forest‐forest plot pairs; (d) Slope‐slope plot pairs; (e) Adjacent forest‐slope plot pairs.

Pairwise comparisons of phylogenetic Sørensen similarity, turnover, and nestedness revealed clear differences among habitat combinations (Figure 8). Forest–forest pairs exhibited the highest phylogenetic Sørensen similarity (0.520), while slope–slope pairs were relatively lower (0.393). The similarity between adjacent slope–forest pairs was intermediate (0.410) but notably higher than that of non‐adjacent slope–forest pairs (0.378). These results suggest that spatial adjacency partially mitigates phylogenetic divergence due to niche differentiation or lineage isolation.

In terms of compositional mechanisms, phylogenetic turnover was the dominant contributor to community dissimilarity across plots, indicating that species lineage replacement primarily shaped phylogenetic differences. However, in adjacent slope–forest pairs, phylogenetic nestedness also played a significant role, suggesting that in geographically proximate habitats, shared lineages and source pool nesting jointly influenced phylogenetic patterns between communities.

**FIGURE 9:**
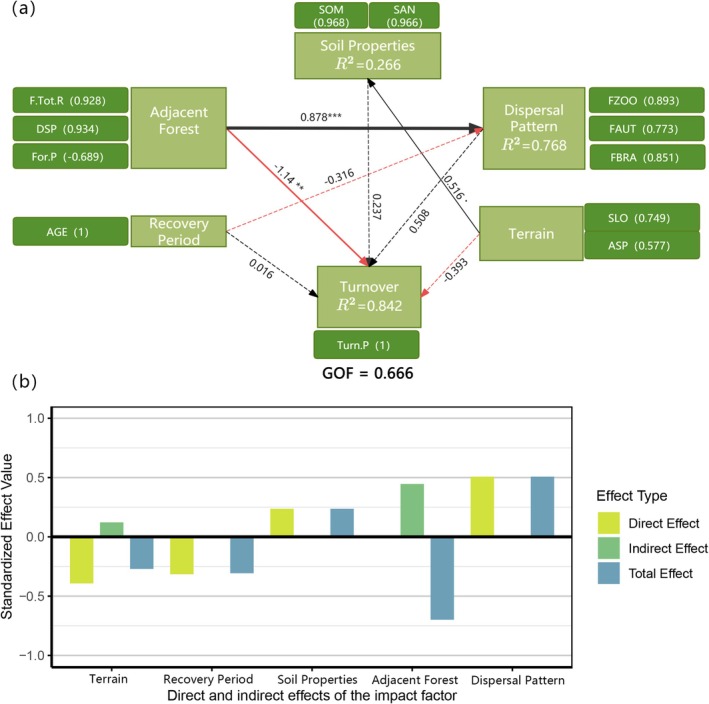
(a) The PLS‐PM model reveals the effects of adjacent forests, soil properties, species dispersal modes, restoration age, topography, and other factors on the phylogenetic turnover between different slopes and forest plots. Black lines indicate positive correlations, while red lines represent negative correlations. Solid lines denote significant relationships with significance levels as follows: ˙*p* < 0.1; *p < 0.05; **p < 0.01; ***p < 0.001. Dashed lines indicate non‐significant relationships. The thickness of the lines reflects the strength of causal relationships, accompanied by standardized path coefficients. (b) Bar chart showing the direct, indirect, and total effects of each factor.

To investigate the drivers of phylogenetic differentiation, we constructed a PLS‐PM structural model linking environmental variables to turnover processes. The model explained 84.2% of the variance in phylogenetic dissimilarity (*R* ^2^ = 0.842; GOF = 0.666; Figure 9). Among all predictors, adjacent forest characteristics were the key determinant. Within this construct, forest species richness (F.Tot.R, loading = 0.928) and distance to the core species pool (DSP, loading = 0.934) were the most influential factors. These findings indicate that propagule pressure and the spatial proximity of adjacent forests play a decisive role in determining the degree of phylogenetic overlap between slope and forest communities.

## 4 Discussion

### 4.1 Composition and Sources of Spontaneous Vegetation on Roadside Slopes

Understanding the composition and sources of naturally regenerated vegetation on roadside slopes is essential for developing effective ecological restoration strategies (Arenas, Escudero, et al. 2017). In this study, we recorded 98 vascular plant species on restored slopes, of which 95.9% were native and only 4.0% were non‐native or invasive species—highlighting a strong dominance of local flora. This pattern suggests that, despite substantial anthropogenic disturbance, the surrounding native species pool continues to serve as a vital propagule source, supporting spontaneous colonization through effective dispersal mechanisms. Notably, we identified three species that, according to regional floristic records, occur exclusively in roadside slope habitats within the study area. Although this pattern may arise through multiple non‐exclusive pathways—including (1) regeneration from residual or persistent soil seed banks within the slope substrate (in situ legacy effects), (2) short‐distance propagule input from adjacent habitats within the surrounding landscape, (3) natural dispersal from the broader regional species pool, and (4) long‐distance or human−vehicle‐mediated propagule input from outside the regional species pool via road‐corridor processes—our occurrence‐based data do not allow us to disentangle the relative contributions of these mechanisms. Nevertheless, the successful establishment of these species suggests that roadside slopes provide environmentally suitable conditions for colonization and persistence, at least with respect to environmental filtering and early‐stage community assembly (Fekete et al. 2017). From a biodiversity conservation perspective, roadside slopes may therefore function not only as interfaces where propagules accumulate, but also as sites where community assembly proceeds under strong abiotic and biotic filters—potentially increasing local species richness while also introducing novel biotic interactions and altering existing community dynamics (Corcos et al. 2020).

In terms of dispersal strategies, our results showed a clear bias toward species with high dispersal efficiency. Wind‐dispersed taxa accounted for 61.2% of slope vegetation, and species exhibiting traits associated with long‐distance dispersal comprised 81.6%—significantly exceeding proportions observed in adjacent forest plots (Campbell et al. 2003). These species, primarily from families such as Rosaceae, Asteraceae, Ericaceae, Poaceae, and Lauraceae, possess diaspores that are small, winged, fleshy, or feathered—traits that enhance dispersal by wind or birds (Wang et al. 2014). These morphological adaptations not only facilitate long‐distance seed transport but may also promote the development of rich and persistent soil seed banks on exposed slopes, thus accelerating spontaneous vegetation recovery (Wang et al. 2020). Interestingly, the distribution of dispersal modes and growth forms among slope species closely mirrored those of species shared between slope and forest plots, while differing substantially from the broader forest species pool (Figure 1). This pattern implies that slope community assembly is shaped not only by propagule availability, but also by post‐dispersal filters, including abiotic constraints and functional trait compatibility—ultimately influencing species recruitment and establishment success (Qin et al. 2023).

### 4.2 The Role of Adjacent Vegetation in Roadside Slope Restoration

Natural recovery on roadside slopes is strongly influenced by landscape context, particularly the presence and characteristics of adjacent vegetation. A growing body of evidence demonstrates that surrounding habitats serve as critical propagule reservoirs for restored sites (Bochet et al. 2007; Kirmer et al. 2008; Catano et al. 2021). In our study, 65.3% of slope species were shared with adjacent forest plots, confirming that nearby forests play a fundamental role in shaping slope vegetation. Phylogenetic similarity between slope and forest communities (41.95%–72.82%) further indicates that restoration outcomes are closely tied to the species pool and structural attributes of adjacent forests, including their area and species richness (Figure 7).

Despite their substantial compositional similarity, slopes and forests differed markedly in demographic structure. On slopes, all non‐tree species were represented by mature reproductive individuals, whereas tree species occurred only in vegetative stages, except for rarely recorded species. In contrast, reproductive individuals of all life forms were observed in adjacent forest stands. This pattern suggests that slopes may not yet support self‐sustaining populations of certain forest tree species and may instead function as demographic sinks for these taxa (Dias 1996). Such immigration‐dependent dynamics are consistent with source–sink processes operating within a metacommunity framework (Mouquet and Loreau 2003). Disruption of functional connectivity through habitat fragmentation could further constrain tree recruitment and alter successional trajectories (Arroyo‐Rodríguez et al. 2013).

Beyond propagule supply, adjacent forests likely enhance restoration success by moderating microclimatic extremes, improving soil conditions through litter input, and facilitating seedling establishment (Remy et al. 2018). These buffering effects may be particularly important for woody species attempting to transition from vegetative to reproductive stages. Consequently, conserving and strategically integrating adjacent forest patches into infrastructure planning should be considered a priority in slope restoration and ecological rehabilitation programs (Le Stradic et al. 2014). Long‐term demographic monitoring will be essential to determine whether current tree populations on slopes can achieve reproductive maturity or remain dependent on external seed sources.

### 4.3 Similarity and Dissimilarity in Species Composition: Influencing Factors

The richness of shared species and the degree of phylogenetic similarity between roadside slopes and adjacent forests are shaped by a suite of interacting ecological factors. Our analysis identified several key predictors, including adjacent forest species richness, proximity to core protected areas, species dispersal capacity, and slope soil physicochemical properties. Among these, forest species richness and proximity to core zones were significantly negatively correlated with phylogenetic dissimilarity (Figures 6 and 7), consistent with previous findings. In species‐rich environments, increased interspecific competition and niche differentiation can limit the success of long‐distance dispersers (Nathan et al. 2008). However, such environments also tend to support extensive regional species pools, ensuring a continuous and diverse propagule supply to both intact forests and nearby disturbed sites (Spasojevic et al. 2018; Jung et al. 2021). In particular, core areas of nature reserves, characterized by intact native vegetation, function as critical source habitats, enhancing seed influx and promoting both taxonomic and phylogenetic convergence between adjacent habitat types.

We also found a significant positive association between forest area and both shared species richness and phylogenetic similarity. This may be due to the role of larger or moderately fragmented forest patches in enhancing edge effects and increasing microhabitat heterogeneity, thereby providing additional ecological niches for colonization. These results align with the “habitat fragmentation promotes diversity” hypothesis proposed by Fahrig (2003), suggesting that fragmentation can sometimes enhance local biodiversity by increasing spatial complexity. Nonetheless, in landscapes with limited forest cover, fragmentation is more likely to exert negative effects by disrupting seed dispersal pathways, constraining species movement, and reducing opportunities for colonization (Rybicki et al. 2020). These contrasting outcomes highlight the scale‐ and context‐dependence of fragmentation effects, underscoring the complex trade‐off between increased connectivity and spatial isolation in shaping species turnover and phylogenetic structure.

## 5 Conclusion

This study demonstrates that spontaneous vegetation recovery on roadside slopes in subtropical mountainous regions is closely linked to adjacent forest communities, both taxonomically and phylogenetically. The predominance of native species with long‐distance dispersal traits highlights the crucial role of ecological connectivity in enabling early‐successional dynamics and shaping species recruitment. Community composition and phylogenetic structure on disturbed slopes are primarily influenced by surrounding vegetation, propagule pressure, and the spatial configuration of nearby forest habitats. These findings underscore the ecological significance of adjacent forests as propagule sources and structural anchors for community assembly. To enhance the effectiveness of passive restoration in linear infrastructure landscapes, conservation planning should prioritize the protection, integration, and spatial connectivity of remnant forest patches. A landscape‐scale approach that facilitates seed flow and reinforces forest–slope linkages will be essential for promoting biodiversity recovery and long‐term ecological resilience in mountainous roadside ecosystems.

## Author Contributions

**Kun‐Rong Qin:** conceptualization (lead), data curation (lead), funding acquisition (lead), investigation (lead), methodology (lead), software (lead), writing – original draft (lead), writing – review and editing (equal). **Zi‐Zhuo Wang:** data curation (equal), investigation (equal), methodology (equal), software (equal). **Feng‐Ping Yang:** funding acquisition (supporting), investigation (equal), methodology (equal). **Xian‐Tao Peng:** investigation (equal), project administration (equal). **Hua Qin:** supervision (equal), writing – review and editing (equal). **Hai‐Yang Wang:** conceptualization (equal), funding acquisition (supporting), writing – review and editing (equal).

## Funding

Kunrong Qin's research was supported by the Talent Introduction and Scientific Research Startup Fund Project of Chongqing College of Humanities, Science & Technology (CRKRC2024004) and the Chongqing National Forest Reserve Effectiveness Monitoring and Scientific Research Experiment Project (No. 2023001121). Fengping Yang's research was supported by the National Natural Science Foundation of China (32101576).

## Conflicts of Interest

The authors declare no conflicts of interest.

## Data Availability

The data supporting the findings of this study are openly available in the Open Science Framework (OSF) repository at https://osf.io/gh26j/?view_only=20c777ef26874bc7b6c8cfdcaeb37276.
